# Estimating the economic burden of colorectal cancer in China, 2019–2030: A population‐level prevalence‐based analysis

**DOI:** 10.1002/cam4.6787

**Published:** 2023-12-19

**Authors:** Hong Wang, Yan‐Jie Li, Lin Lei, Cheng‐Cheng Liu, Wan‐Qing Chen, Min Dai, Xin Wang, Jie‐Bin Lew, Ju‐Fang Shi, Ni Li, Jie He

**Affiliations:** ^1^ Office of Cancer Screening, National Cancer Center/National Clinical Research Center for Cancer/Cancer Hospital Chinese Academy of Medical Sciences and Peking Union Medical College Beijing China; ^2^ Department of Cancer Epidemiology The Affiliated Cancer Hospital of Zhengzhou University & Henan Cancer Hospital Zhengzhou China; ^3^ Department of Cancer Control and Prevention Shenzhen Center for Chronic Disease Control Shenzhen China; ^4^ Chinese Academy of Medical Sciences Key Laboratory for National Cancer Big Data Analysis and Implement Chinese Academy of Medical Sciences and Peking Union Medical College Beijing China; ^5^ Department of Cancer Epidemiology, National Cancer Center/National Clinical Research Center for Cancer/Cancer Hospital Chinese Academy of Medical Sciences and Peking Union Medical College Beijing China; ^6^ The Daffodil Centre The University of Sydney, a Joint Venture with Cancer Council New South Wales Sydney Australia; ^7^ Department of Thoracic Surgery, National Cancer Center/National Clinical Research Center for Cancer/Cancer Hospital Chinese Academy of Medical Sciences and Peking Union Medical College Beijing China

**Keywords:** China, colorectal cancer, costs, economic burden, population‐level

## Abstract

**Background:**

Colorectal cancer (CRC) is one of the most common cancers worldwide. Comprehensive data on the economic burden of CRC at a population‐level is critical in informing policymaking, but such data are currently limited in China.

**Methods:**

From a societal perspective, the economic burden of CRC in 2019 was estimated, including direct medical and nonmedical expenditure, disability, and premature‐death‐related indirect expenditure. Data on disease burden was taken from the GBD 2019 and analyzed using a prevalence‐based approach. The per‐person direct expenditure and work loss days were from a multicenter study; the premature‐death‐related expenditure was estimated using a human capital approach. Projections were conducted in different simulated scenarios. All expenditure data were in Chinese Yuan (CNY) and discounted to 2019.

**Results:**

In 2019, the estimated overall economic burden of CRC in China was CNY170.5 billion (0.189% of the local GDP). The direct expenditure was CNY106.4 billion (62.4% of the total economic burden), 91.4% of which was a direct medical expenditure. The indirect expenditure was CNY64.1 billion, of which 63.7% was related to premature death. The predicted burden would reach CNY560.0 billion in 2030 given constant trends for disease burden; however, it would be alternatively reduced to <CNY515.2 billion if the cancer prevention and control goals set by the United Nations and China for 2030 are achieved.

**Conclusions:**

The population‐level economic burden of CRC in China in 2019 seemed noteworthy, with the direct expenditure accounting for more than half. Without effectively reducing exposure to modifiable factors and expanding screening coverage, the burden would continue increasing.

## 1 INTRODUCTION

Colorectal cancer (CRC) is one of the most common cancers both globally and in China. The Global Burden of Disease Study (GBD) 2019 reported that CRC was the second most prevalent cancer in China in 2019, with Chinese patients accounting for an estimated 30% of the global CRC patient population.^1^ In addition, treatment of CRC is costly, and the per‐person direct medical expenditure was estimated at 61,829 Chinese Yuan (CNY) in 2014 and has doubled over one decade.^2^, ^3^ The substantial number of patients with CRC translates to a heavy economic burden on society. Comprehensive data on the economic burden of CRC is critical in informing policymaking.

The population (national) level economic burden of cancer has already been estimated and predicted in many countries.^4^, ^5^, ^6^, ^7^, ^8^, ^9^, ^10^, ^11^, ^12^, ^13^ However, there are currently few comprehensive data available for China. Most of the previous studies focus only on the per‐person direct medical expenditure related with CRC diagnosis and treatment alone.^2^, ^14^, ^15^, ^16^, ^17^, ^18^, ^19^, ^20^ Few studies have estimated the direct nonmedical cost and indirect costs of CRC.^3^, ^14^, ^21^

This study attempted to estimate the economic burden of CRC at the national‐level in China in 2019 and predict the annual economic burden in 2020 and 2030 based on the predicted epidemiological and demographic data as well as cancer prevention and control goals set by the United Nations and China in 2030.^22^, ^23^ The results will inform CRC prevention and control, and associated budget planning in China. Furthermore, the methodology will provide references for other countries to estimate the population‐level economic burden of cancers and other chronic diseases.

## 2 MATERIALS AND METHODS

### 2.1 Study design

**FIGURE 1:**
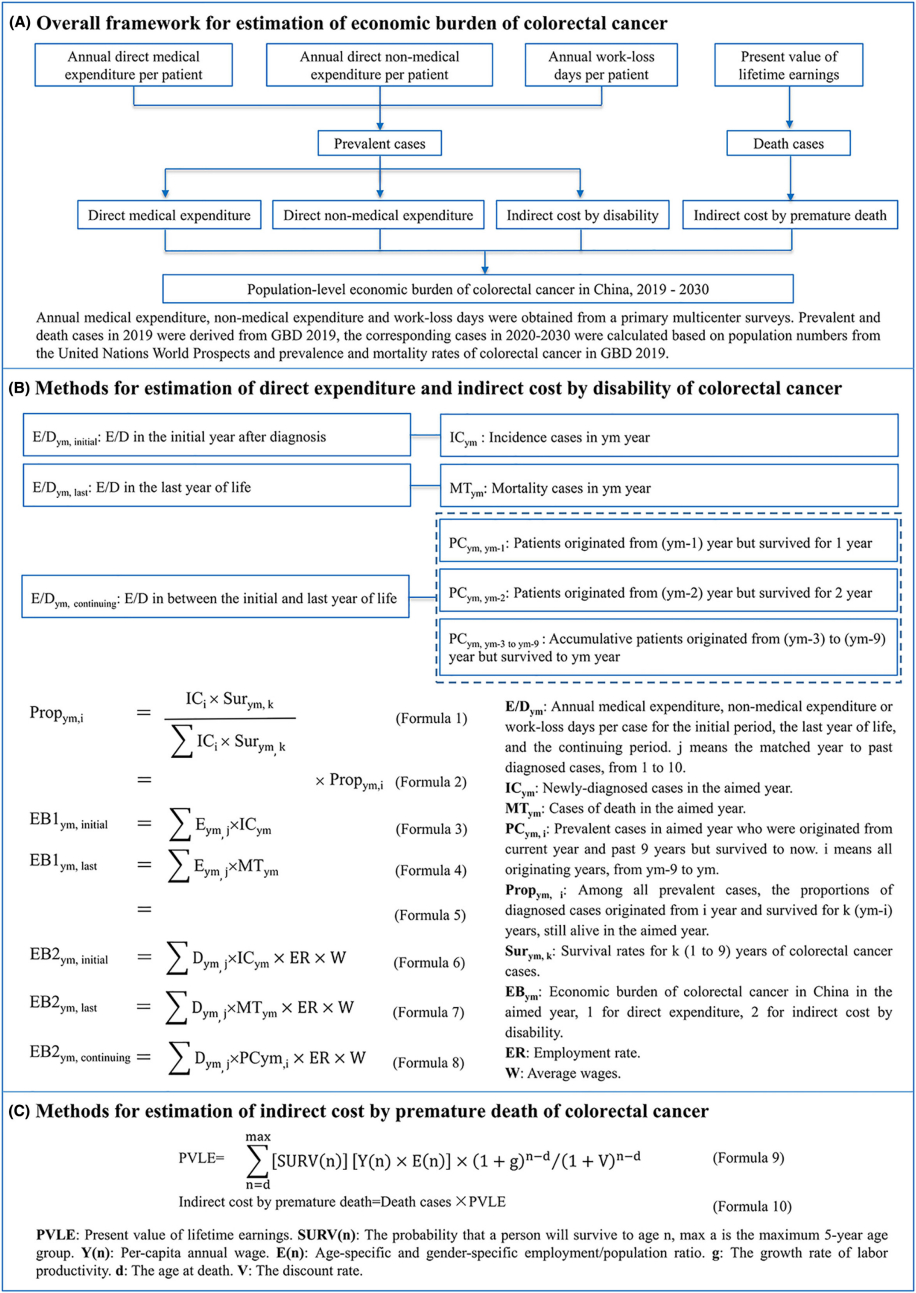
Overall study design.

From a societal perspective, a prevalence‐based approach, which has previously been used to assess the economic burden of non‐communicable diseases, was used to estimate the annual economic burden of CRC at the national level for the years 2019 to 2030.^24^, ^25^ The overall cost estimation framework is displayed in Figure 1A.

### 2.2 Data sources

The annual averages for direct medical, nonmedical expenditure, and work loss days were obtained from a hospital‐based multicenter survey from 13 provinces across China.^3^ The age‐ and sex‐specific data for CRC disease burden in 2019 was obtained from the GBD 2019 (Table S1).^1^ The one‐to‐ten‐year survival rates of CRC patients of different age groups and gender were derived from the National Cancer Registry and other population‐based studies,^26^, ^27^ and these survival rates were used to calculate the proportion of prevalent patients in 2019 derived from each year in the past 10 years of 2019. The age and sex‐specific survival probability of the general population were derived from the 2016 life tables of the Chinese population reported by the World Health Organization,^28^ which is used to estimate the economic burden caused by the premature death of CRC patients. Based on the annual average wage in 2018 reported in the China Statistical Yearbook 2019,^29^ the annual average wage for 2019–2030 was estimated according to the growth rate of China's GDP predicted by the OECD (Organization for Economic Co‐operation and Development).^30^ The employment rate was derived from the Chinese employment rate in 2010.^31^ Future population data were obtained from the United Nations World Population Prospects 2019^32^ (Table S2). All data in this study is adapted by public databases and other literature, and therefore does not necessitate the consent of a local ethics committee.

### 2.3 Baseline analysis

#### 2.3.1 Direct expenditure and disability‐related indirect expenditure

This study assumed that the prevalent CRC patient in 2019 comprised patients newly diagnosed in 2019 and patients who survived after being diagnosed over the last 9 years. The annual medical expenditure, nonmedical expenditure, and work loss days of patients over the whole disease course were decomposed by each consecutive 12‐month period after the CRC was diagnosed.^24^, ^33^ To ensure the data robustness, only those by‐year sub‐groups with a minimum sample size of 50 were included, and therefore the annual averages in the first year, the second year, and the third year after the CRC diagnosis were calculated. The detailed annual average expenditure and work loss days of CRC patients were shown in Table S3.

As there were no relevant reports on the whole course expenditure patterns of CRC patients in China, detailed information was available for CRC patients in the US^8^; therefore, we applied the by‐year pattern to estimate the expenditure for the fourth year to the tenth year after CRC diagnosed. The US National Cancer Institute's Cancer Prevalence and Cost of Care Projections divides patients with cancer into three phases according to their disease course: the “initial phase” (the first year after diagnosis), the “last year of life phase” (the last year before death), and the “continuing phase” (the period between the initial phase and the last year of life phases)^8^; the ratios of medical expenditure of CRC patients (2018) in the initial phase, continuing, and the last year of life phase was reported as 1: 0.07: 0.78, respectively.^34^ Using these ratios among phases, we leveraged our existing expenditure data sets of the first year to the third year to estimate the data sets for the unknown fourth year to the tenth year.

At the same time, CRC patients in 2019 were categorized into three phases: the initial phase (newly diagnosed cases), the last year of life phase (patients who died during the year), and the continuous phase (all other patients). The number of CRC patients in the three phases by subgroups was estimated according to the proportion of different regions and CRC stages investigated in the above cross‐sectional study,^3^ the proportion of different cancer locations occurred reported in the Chinese Cancer Registry Annual Report,^35^ the overall annual incident CRC cases in GBD 2019,^1^ as well as the one‐to‐ten‐year survival rates of CRC patients estimated according to the literature.^26^, ^27^ The direct and indirect economic burden of CRC was calculated similarly in detail in Figure 1B. Since the unavailability of detailed wages of different subgroups, the overall daily average wage was used to calculate the indirect economic burden caused by disability in different subgroups of CRC patients. All expenditure data were in 2019 currency and were discounted by 3% per year, as well as an annual average growth rate of 6.9%.^14^

#### 2.3.2 Indirect expenditure caused by premature death

The indirect expenditure due to premature deaths were estimated for CRC patients between the age of 15 years old (working age) and the average life expectancy (male: 74.5 years old, female: 80.0 years old).^1^ First, the present value of lifetime earnings (PVLE) for each 5‐year age group in 2019 was estimated based on the human capital approach.^36^ Future earnings from 2020 to average life expectancy were inflated by 8.7% per year to reflect the annual productivity growth rate based on the observed annual GDP growth rate in 2013–2018^29^ and were discounted to 2019 at a rate of 3% per year. Second, to estimate the indirect economic burden caused by premature death due to CRC, the number of deaths of CRC patients in each 5‐year age group in 2019 was multiplied by the PVLE of each five‐year age group in 2019 calculated above (Figure 1C).

### 2.4 Prediction for up to 2030

The disease burden of CRC from 2020 to 2030 was predicted in four scenarios: (1) Demographic change: considering population growth alone, with the rates of incidence, prevalence, and mortality of CRC remaining unchanged. (2) Base case: considering the trend of population growth, along with the trends of CRC rates of incidence, mortality, and prevalence according to the rates from 2009 to 2019 reported in the GBD 2019. (3) Sustainable Development Goals (SDGs) 2030: reducing the premature mortality rate of non‐communicable diseases by one‐third in 2030 compared with 2015.^22^ In this study, non‐communicable diseases are referred to as CRC, and 2019 was taken as the base year. The SDGs 2030 (a) considers only the decline in mortality and (b) considers the interrelationship between mortality, incidence, and prevalence—these three indicators are expected to decrease by one‐third simultaneously. (4) Healthy China 2030: increasing the five‐year survival rate of all patients with cancer by 15%^23^; for this study, only CRC was considered.

The average direct medical expenditure and nonmedical expenditure were predicted for the corresponding years based on the average annual growth rate of 6.9%, and the number of work loss days remained unchanged.

### 2.5 Sensitivity analysis

One‐way sensitivity analysis was used to explore the uncertainty of the economic burden of CRC in China from 2019 to 2030. The impact of the following factors was considered: the annual growth rate of direct expenditure, consultation rate, annual growth rate of productivity and/or earnings, working age, prevalence breakdown, and prevalence, incidence, and mortality data source. The relative change rate of the total economic burden under the base case scenario was used to measure the impact of the above variations (Data S1).

## 3 RESULTS

### 3.1 Economic burden estimation for 2019

The total economic burden of CRC in China in 2019 was estimated to be CNY170.5,^29^ and when the OECD‐reported GDP of China in 2019^30^ was adopted, the proportion was 0.105%. The direct economic burden of CRC was estimated to be CNY106.4 billion (62.4% of the total economic burden), which included direct medical expenditure of CNY97.3 billion (approximately 1.65% of the total health expenditure in China in 2019^37^) and direct nonmedical expenditure of CNY9.1 billion. Indirect costs were estimated to amount to CNY64.1 billion, with CNY23.3 billion attributed to disability and CNY40.9 billion to premature death, respectively (Table 1).

**TABLE 1:** Estimated population‐level economic burden of colorectal cancer in China in 2019, overall and by subgroup.

Overall and subgroups	Direct expenditure				Indirect expenditure			The overall economic burden			
	Medical expenditure, CNY billion	Nonmedical expenditure, CNY billion	Sub‐total, CNY billion	The^a^ percent, %	Disability‐related, CNY billion	Premature death related, CNY billion	Sub‐total, CNY billion	Total		GDP^c^ percent %	
								CNY billion	USD^b^ billion	China^d^	OECD^e^
Overall	97.3	9.1	106.4	1.80	23.3	40.9	64.1	170.5	24.7	0.189	0.105
Age at diagnosis, years											
<45	9.9	0.9	10.8	0.18	2.8	18.1	20.9	31.7	4.6	0.035	0.019
45–59	25.6	2.4	27.9	0.47	6.4	16.9	23.3	51.2	7.4	0.057	0.031
≥60	61.8	5.8	67.6	1.14	14.1	5.9	20.0	87.6	12.7	0.097	0.054
Gender											
Male	59.7	5.6	65.3	1.10	14.4	30.3	44.7	110.0	15.9	0.122	0.068
Female	37.6	3.5	41.1	0.69	8.8	10.6	19.5	60.5	8.8	0.067	0.037
Region											
East	44.7	4.5	49.3	0.83	10.6	18.7	29.4	78.6	11.4	0.087	0.048
Central	27.8	2.6	30.4	0.51	7.7	11.1	18.7	49.1	7.1	0.055	0.030
West	24.8	1.9	26.7	0.45	5.0	11.1	16.1	42.8	6.2	0.047	0.026
Cancer stage											
I	11.5	1.3	12.8	0.22	2.5	5.8	8.2	21.0	3.0	0.023	0.013
II	23.5	1.7	25.2	0.43	3.9	11.1	15.0	40.2	5.8	0.045	0.025
III	34.5	3.6	38.1	0.64	8.9	14.3	23.2	61.3	8.9	0.068	0.038
IV	27.8	2.5	30.2	0.51	8.0	9.8	17.8	48.0	7.0	0.053	0.029
Location of cancer occurred											
Colon	48.2	4.5	52.7	0.89	11.5	20.0	31.5	84.3	12.2	0.094	0.052
Rectum	47.9	4.5	52.3	0.89	11.5	20.4	31.8	84.2	12.2	0.094	0.052
Anus	1.2	0.1	1.3	0.02	0.3	0.5	0.8	2.1	0.3	0.002	0.001

^a^ Total health expenditure in China, 2019.

^b^ United States dollars, 1 USD = 6.90 CNY.

^c^ Gross domestic productivity in China, 2019.

^d^ GDP from China Statistical Year Book 2019.

^e^ GDP of China from Organization for Economic Co‐operation and Development.

**FIGURE 2:**
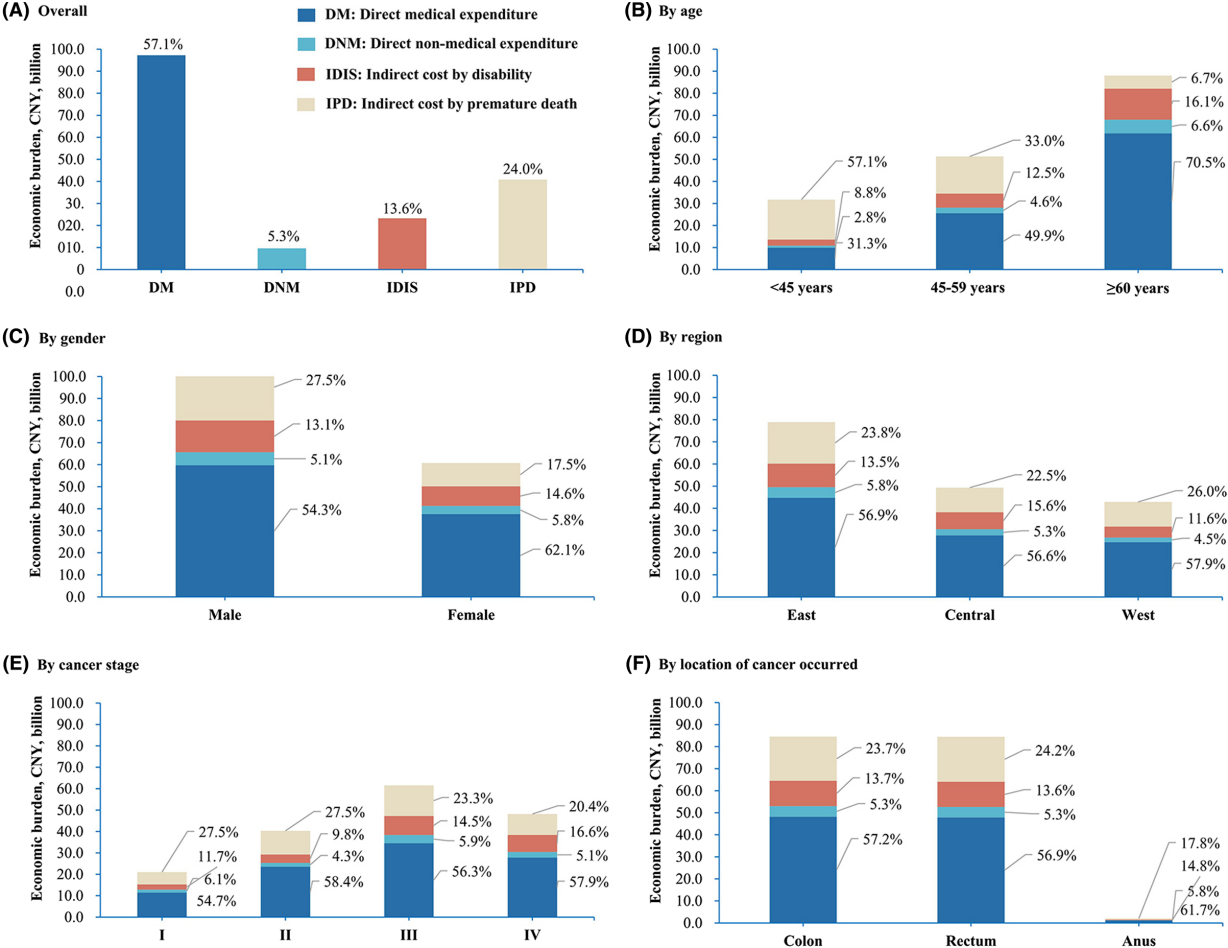
A breakdown of the population‐level economic burden of colorectal cancer in China in 2019, by subgroup.

Patients over 60 years old (51.4%), male patients (64.5%), those living in the eastern region of China (46.1%), those with advanced‐stage CRC (Stages III and IV) (64.1%), and those with colon cancer or rectal cancer (98%) accounted for the majority of the economic burden associated with CRC. The direct medical burden, direct nonmedical burden, the indirect economic burden caused by disability, and the indirect economic burden caused by premature death accounted for different proportions across different subgroups (Figure 2).

### 3.2 Economic burden prediction for 2020, 2025, and 2030

**FIGURE 3:**
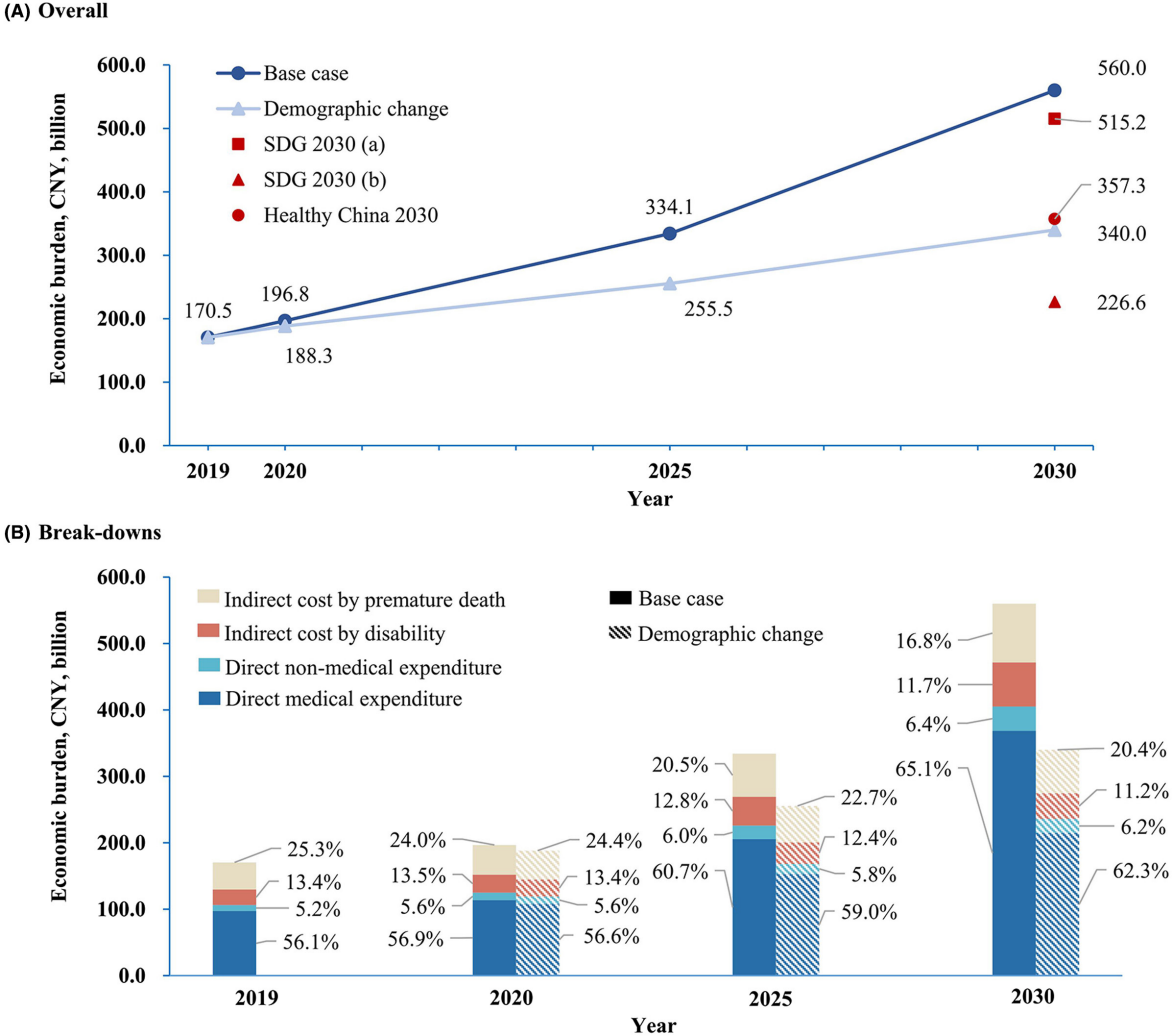
Total and breakdown of the estimated population‐level economic burden of colorectal cancer in China, 2019–2030.

If demographic change alone is considered, the economic burden of CRC in China would increase by 10.5%, 49.9%, and 99.4%, respectively, compared with 2019. Under the base case scenario, the economic burden of CRC in China would increase by 15.4%, 95.9%, and 228.4%, respectively, compared with 2019. Regarding the composition of economic burden, the proportion of direct medical burden may increase, while the proportion of indirect economic burden caused by premature death may decrease (Figure 3).

If the SDGs were to be achieved by considering the reduction of premature death from CRC, together with a similar reduction in the prevalence and incidence, the economic burden of CRC would drop to CNY226.6 million by 2030, with a reduction of 59.5% when compared to the base case scenario. Even if only the decline in premature death were considered, the economic burden would drop to CNY515.2 billion. Further, if the goal of Healthy China 2030 were to be achieved, the economic burden of CRC would be CNY357.3 billion, which is 36.2% lower than the figure in the base case scenario (Figure 3).

### 3.3 Sensitivity analysis

**FIGURE 4:**
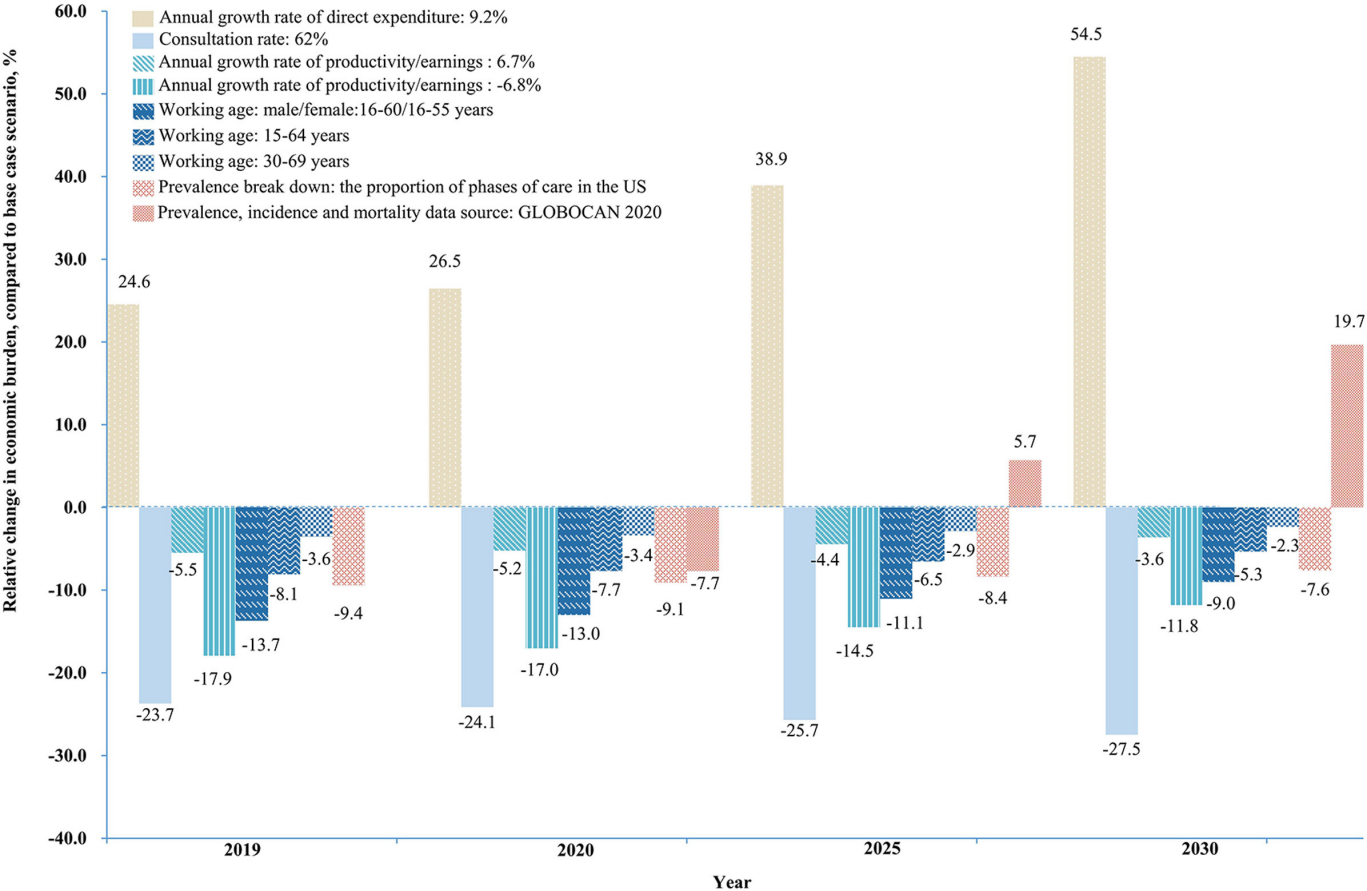
Sensitivity analyses for the estimated population‐level economic burden of colorectal cancer in China, 2019–2030.

The annual growth rate of direct expenditure was found to have the greatest impact on the economic burden, and an annual growth rate of 9.2% would see the total economic burden of CRC in 2019, 2020, 2025, and 2030 increase by 24.6%, 26.5%, 38.9%, and 54.5%, respectively, compared with the baseline. The economic burden can also be easily influenced by the consultation rate. Assuming a lower consultation rate (62%), the economic burden would be reduced by 23%–28%. The economic burden is also affected by the annual productivity growth rate, which is determined by the economic environment. It would be reduced by 3%–6% using a conservative estimate (annual productivity growth rate: 6.7%) and by almost 10%–18% using the worst‐case scenario (−6.8%). With a working age of 16–60 years for men and 16–55 years for women, the economic burden would be reduced by between 9% and 14%, and with a working age of 15–64 years or 30–69 years, the economic burden would decrease by less than 10% compared with baseline. If the number of patients with CRC was divided according to the three clinically relevant phases defined by the United States National Cancer Institute, the economic burden would be reduced by between 7% and 10%. Finally, if the disease burden of CRC in GLOBOCAN 2020 were used, the economic burden would decrease by 7.7% compared with the baseline in 2020, and increase by 5.7% and 19.7% compared with the baseline in 2025 and 2030, respectively (Figure 4).

## 4 DISCUSSION

We estimated the national‐level economic burden of CRC for the period from 2019 to 2030 in China using a prevalence‐based approach. To the best of our knowledge, this is the most comprehensive population‐level analysis of the economic burden of CRC in China. The economic burden of CRC in China was estimated to be CNY170.5 billion in 2019, of which the direct economic burden accounted for 62.4%. Moreover, without effective intervention against the growing disease burden and treatment cost of CRC, the economic burden may continue to increase over the next 10 years.

This study estimated the economic burden of CRC from a social perspective, including direct medical expenditure, direct nonmedical expenditure, and indirect economic burden. Previously, there were few studies reported the indirect economic burden of CRC patients at the national‐level in China. One study based on the Chinese hospital database, estimated that the direct medical expenditure on CRC in China was CNY20.76 billion in 2015,^17^ which is considerably lower than the estimate in our study. This difference may be because the disease burden of CRC increased in the years from 2015 to 2019. Another reason may be that the study mentioned was based on the Chinese hospital database and did not include patients who receive treatment in hospitals below second‐level, which might have led to an underestimation of the direct medical economic burden.

In this study, the proportion of direct expenditure of CRC in the total economic burden was higher than that of indirect economic burden. Cancers with different prognosis have different proportions of the direct and indirect economic burden. Those with higher survival rates live longer with tumors, resulting in relatively high direct expenditure. The survival rate of patients with CRC is relatively high among all cancers.^26^ Consequently, the direct economic burden constituted a larger proportion of the total economic burden. In contrast, for cancers with poor survival rates, such as lung cancer, the indirect economic burden constitutes a larger proportion of the overall economic burden.^25^

Predicting the future economic burden of CRC is important for policymaking and medical resource allocation. Considering different scenarios, this study predicted the economic burden of CRC between 2020 and 2030. Even if the disease burden of CRC remains unchanged, the economic burden will continue to increase due to population aging and urbanization and is expected to double between 2019 and 2030. If the SDGs 2030 and Healthy China 2030 goals are achieved by 2030, the economic burden of CRC would be greatly reduced. These results highlight the urgency of introducing interventions to reduce CRC incidence and mortality.

Individual‐level of medical expenses has a considerable impact on the estimation of the overall economic burden. The direct expenditure per case in this study came from a multicenter cross‐sectional survey, and only the expenditure in the first 3 years post‐diagnosis was recorded. To obtain the expenditure for different periods throughout the course of the disease, we estimated the expenditure in the continuing and last year of life phases according to the cost ratio of the three phases of CRC in the USA. However, the cost patterns of CRC vary between countries.^33^, ^34^ No study has reported on the cost patterns of CRC in China. This study made a preliminary estimation based on the estimated ratio in the USA, which was relatively conservative compared with the proportions in Australia. We expect that there will be studies on the cost patterns of the entire course of CRC in China in the future to facilitate a more accurate estimation of the economic burden.

This study has limitations. First, the direct cost per case came from a cross‐sectional survey undertaken between 2012 and 2014. Although the cost was discounted according to the annual growth rate, it still may not represent the actual cost in the estimated year. Second, due to the limitations of the data source available, only total (not by sub‐groups) numbers of prevalent CRC patients were available. Third, in the sensitivity analysis of consultation rate, as the parameters of the impact of consultation rate on survival rate cannot be obtained, we only consider the impact of consultation rate on direct medical and nonmedical expenditure, which may have a certain impact on the overall results. However, despite the above limitations, by predicting the economic burden of CRC at the population‐level in China, this study fills a gap in the literature and will provide a basis for the formulation of relevant health policies.

## 5 CONCLUSIONS

In summary, the economic burden of CRC in China in 2019 is considerable, and the direct economic burden accounted for a higher proportion of the total economic burden compared to cancers with a poor prognosis, such as lung cancer. Without the intervention of modifiable risk factors and the expansion of effective screening, the economic burden of CRC will continue to increase over the next decade. Targeted intervention measures should be strengthened to decrease this trend. Despite the study's limitations, its estimation of the current economic burden of CRC and predictions of future trends will provide a basis for the formulation of cancer prevention and control policies, and resource allocation decisions in the future.

## AUTHOR CONTRIBUTIONS

**Hong Wang:** Data curation (equal); formal analysis (lead); methodology (equal); validation (equal); visualization (equal); writing – original draft (lead); writing – review and editing (equal). **Yan‐Jie Li:** Data curation (equal); formal analysis (equal); methodology (equal); validation (equal); visualization (equal); writing – original draft (equal); writing – review and editing (equal). **Lin Lei:** Data curation (supporting); formal analysis (supporting); methodology (supporting); validation (equal); visualization (supporting); writing – original draft (equal); writing – review and editing (equal). **Cheng‐Cheng Liu:** Data curation (supporting); formal analysis (supporting); methodology (equal); visualization (supporting); writing – review and editing (supporting). **Wan‐Qing Chen:** Conceptualization (supporting); methodology (supporting); supervision (supporting); writing – review and editing (supporting). **Min Dai:** Conceptualization (supporting); methodology (supporting); supervision (supporting); writing – review and editing (supporting). **Xin Wang:** Data curation (supporting); writing – review and editing (supporting). **Jie‐Bin Lew:** Supervision (supporting); writing – review and editing (supporting). **Ju‐Fang Shi:** Conceptualization (lead); funding acquisition (equal); methodology (equal); supervision (equal); writing – review and editing (lead). **Ni Li:** Conceptualization (equal); funding acquisition (equal); supervision (equal); writing – review and editing (equal). **Jie He:** Conceptualization (equal); supervision (equal); writing – review and editing (equal).

## FUNDING INFORMATION

National Natural Science Foundation of China (81773521); The Non‐profit Central Research Institute Fund of Chinese Academy of Medical Sciences (2019PT320027); An Open Competition Grant from Health Policy and System Sciences of China Medical Board (19–340); A Talent Incentive Plan Sponsored by Cancer Hospital, Chinese Academy of Medical Sciences.

## CONFLICT OF INTEREST STATEMENT

We declare no conflict of interest.

## Supporting information


Data S1.

## Data Availability

The authors confirm that the data supporting the findings of this study are available within the article and its supplementary materials.
