# Supplementary material for: Estimating the economic burden of colorectal cancer in China, 2019–2030: A population‐level prevalence‐based analysis
Source: Cancer Med. 2023 Dec 19;13(1):e6787. doi: 10.1002/cam4.6787 (PMC10807552; doi:10.1002/cam4.6787)
Supplement: Supplementary file 1 — Data S1. [file CAM4-13-e6787-s001.docx]

**Supplemental Online Content**

This appendix provides more supplemental results for "*Estimating the economic burden of colorectal cancer in China, 2019-2030: a population-level prevalence-based analysis*”

Data S1. The detailed method of one-way sensitivity analysis of the economic burden of CRC in China from 2019 to 2030.

Table S1. Inputting parameters of diseases burden of colorectal cancer and population numbers in China

Table S2. Inputting parameters of survival probability, employment rates, and number of populations in China

Table S3. Average annual direct expenditure and work loss days per colorectal cancer patient in China in 2019, by year post-diagnosis

**Data S1. The detailed method of one-way sensitivity analysis of the economic burden of CRC in China from 2019 to 2030.**

In one-way sensitivity analysis, we considered the impact of the following factors: (1) The annual growth rate of direct expenditure: According to a multicenter hospital-based retrospective clinical epidemiological survey, the annual growth rate of the direct medical expenditure of patients with CRC was 9.2% (Ref: Shi J, et al. Chinese journal of cancer research 2019; 31(5): 825-37.). A growth rate of 9.2% was therefore used to explore the impact on the economic burden. (2) Consultation rate: In the baseline analysis, the assumption was made that all patients with CRC receive treatment. However, according to the fifth National Health Service Survey, 38% of patients do not visit a hospital (Ref: Cai Y, et al. Chinese journal of cancer research 2017; 29(3): 253-62; Center for Health Statistics and Information, NHFPC. An Analysis Report of National Health Services Survey in China, 2013. Beijing: Peking Union Medical College Press, 2016.). On this basis, a consultation rate of 62% was used to explore the potential impact, but here we only considered the impact on direct medical and non-medical expenditure. (3) Annual growth rate of productivity and/or earnings: Productivity can be easily affected by the economic environment. Therefore, this study made two assumptions about the annual growth rate of productivity. First, according to the OECD-reported annual growth rate of China's GDP from 2014 to 2019, an annual growth rate of 6.7% was used as a conservative estimate (Organization for Economic Co-operation and Development. GDP long-term forecast. https://data.oecd.org/gdp/gdp-longterm-forecast.htm.). The worst productivity growth rate of -6.8% was also explored based on China's productivity growth rate of GDP in the first quarter of 2020 during the COVID-19 pandemic (National Bureau of Statistics of China. Preliminary results for GDP in the first quarter of 2020. http://www.stats.gov.cn/xxgk/sjfb/zxfb2020/202004/t20200418_1767701.html.). (4) Working age: The impact of three recommended ranges of working age was explored, including: (a) 16 to 60 years for males and 15 to 55 years for females in line with Chinese regulations (Wu ZG. Dictionary of Population Sciences. Chengdu, Southwestern University of Finance and Economics Press, 1997.); (b) 15 to 64 years, as suggested by the OECD (Organization for Economic Co-operation and Development. Working age population. https://data.oecd.org/pop/working-age-population.htm.); and (c) 30 to 69 years, as this was the best available approximation for working-age cohorts (Goodchild M, et.al. Tobacco control 2018; 27(1):58-64.). (5) Prevalence breakdown: subdivision according to the proportion of patients in three phases of disease (initial, continuing, and last year of life phase) in the USA (National Institutes of Health. Cancer prevalence and cost of care projections. https://costprojections.cancer.gov/graph.php.). (6) Prevalence, incidence, and mortality data source: the disease burden of CRC in GLOBOCAN 2020 was used to test the potential impact of the data source on the results (Ferlay J et al. Global Cancer Observatory: Cancer Tomorrow. Lyon, France: International Agency for Research on Cancer. https://gco.iarc.fr/tomorrow.).

**Table S1 Inputting parameters of diseases burden of colorectal cancer and population numbers in China**

|  | | **Number of cases** | | | | | | | |  | **Rates, per 100 000 population** | | | | | | | | | |
| --- | --- | --- | --- | --- | --- | --- | --- | --- | --- | --- | --- | --- | --- | --- | --- | --- | --- | --- | --- | --- |
|  |  | **New cases** | |  | **Deaths** | |  | **Prevalent cases** | |  | **Incidence** | | |  | **Mortality** | | |  | **Prevalence** | |
|  |  | **Male** | **Female** |  | **Male** | **Female** |  | **Male** | **Female** |  | **Male** | | **Female** |  | **Male** | | **Female** |  | **Male** | **Female** |
| Total | | 433,348 | 249,530 |  | 196,574 | 122,629 |  | 2,328,383 | 1,337,104 |  | 53·8 | | 31·2 |  | 22·7 | | 13·9 |  | 302·2 | 176·8 |
| By age group, years | |  |  |  |  |  |  |  |  |  |  | |  |  |  | |  |  |  |  |
|  | 5-9 | 47 | 34 |  | 12 | 9 |  | 335 | 220 |  | 0·1 | 0·1 | |  | 0·0 | 0·0 | |  | 0·9 | 0·7 |
|  | 10-14 | 48 | 39 |  | 14 | 11 |  | 331 | 253 |  | 0·1 | 0·1 | |  | 0·0 | 0·0 | |  | 0·9 | 0·8 |
|  | 15-19 | 439 | 227 |  | 101 | 52 |  | 3,179 | 1,531 |  | 1·1 | 0·6 | |  | 0·3 | 0·1 | |  | 7·9 | 4·4 |
|  | 20-24 | 1,178 | 574 |  | 293 | 143 |  | 8,480 | 3,969 |  | 2·8 | 1·5 | |  | 0·7 | 0·4 | |  | 19·8 | 10·2 |
|  | 25-29 | 2,979 | 1,392 |  | 786 | 373 |  | 20,967 | 9,761 |  | 5·3 | 2·6 | |  | 1·4 | 0·7 | |  | 37·2 | 18·0 |
|  | 30-34 | 7,264 | 2,878 |  | 2,117 | 852 |  | 49,574 | 19,576 |  | 11·1 | 4·5 | |  | 3·2 | 1·3 | |  | 75·9 | 30·7 |
|  | 35-39 | 10,080 | 3,900 |  | 2,839 | 1,130 |  | 69,196 | 26,890 |  | 19·6 | 7·9 | |  | 5·5 | 2·3 | |  | 134·6 | 54·3 |
|  | 40-44 | 15,538 | 6,441 |  | 4,563 | 1,929 |  | 104,989 | 44,044 |  | 30·0 | 12·9 | |  | 8·8 | 3·9 | |  | 202·4 | 88·5 |
|  | 45-49 | 24,209 | 10,284 |  | 7,781 | 3,316 |  | 160,106 | 68,541 |  | 39·2 | 17·3 | |  | 12·6 | 5·6 | |  | 258·9 | 115·1 |
|  | 50-54 | 37,686 | 18,395 |  | 12,186 | 5,836 |  | 244,009 | 121,719 |  | 60·0 | 29·5 | |  | 19·4 | 9·4 | |  | 388·3 | 195·5 |
|  | 55-59 | 41,934 | 21,639 |  | 14,134 | 7,113 |  | 267,838 | 142,060 |  | 88·0 | 45·8 | |  | 29·7 | 15·1 | |  | 562·2 | 301·0 |
|  | 60-64 | 49,368 | 26,917 |  | 17,828 | 9,413 |  | 303,075 | 171,833 |  | 125·1 | 68·9 | |  | 45·2 | 24·1 | |  | 768·0 | 439·6 |
|  | 65-69 | 59,395 | 34,101 |  | 23,232 | 12,933 |  | 341,681 | 206,923 |  | 171·9 | 95·2 | |  | 67·2 | 36·1 | |  | 989·0 | 577·4 |
|  | 70-74 | 55,214 | 33,625 |  | 24,726 | 14,698 |  | 289,539 | 188,327 |  | 236·7 | 137·1 | |  | 106·0 | 59·9 | |  | 1,241·2 | 767·8 |
|  | 75-79 | 41,681 | 25,417 |  | 22,273 | 13,658 |  | 189,443 | 123,459 |  | 294·8 | 161·8 | |  | 157·6 | 86·9 | |  | 1,340·1 | 785·9 |
|  | ≥80 | 43,144 | 31,833 |  | 31,844 | 25,582 |  | 137,820 | 103,998 |  | 366·4 | 172·2 | |  | 270·4 | 138·4 | |  | 1,170·4 | 562·5 |

**Table S2** Inputting parameters of survival probability, employment rates, and number of populations in China

| **Characteristics** | | **Survival probability** | |  | **Employment rate, %** | |  | **Number of populations** | | | | | | | |
| --- | --- | --- | --- | --- | --- | --- | --- | --- | --- | --- | --- | --- | --- | --- | --- |
|  |  |  |  |  |  |  |  | **Year 2020** | |  | **Year 2025** | |  | **Year 2030** | |
|  |  | **Male** | **Female** |  | **Male** | **Female** |  | **Male** | **Female** |  | **Male** | **Female** |  | **Male** | **Female** |
| Total | | NA | NA |  | 76·1 | 61·7 |  | 738,247,340 | 701,076,434 |  | 746,460,564 | 711,447,684 |  | 748,158,670 | 716,181,480 |
| By age group, years | | | | | | | | | | | | | | | |
|  | 0-4 | <1: 0·992 1-4: 0·999 | <1: 0·992 1-4: 0·999 |  | NA | NA |  | 44,456,332 | 39,476,105 |  | 40,107,551 | 36,234,058 |  | 37,006,608 | 34,026,171 |
|  | 5-9 | 0·998 | 0·998 |  | NA | NA |  | 46,320,144 | 40,415,039 |  | 44,354,672 | 39,398,851 |  | 40,024,719 | 36,167,828 |
|  | 10-14 | 0·999 | 0·999 |  | NA | NA |  | 45,349,923 | 38,912,828 |  | 46,238,892 | 40,353,824 |  | 44,284,584 | 39,344,410 |
|  | 15-19 | 0·998 | 0·998 |  | 32·1 | 29·7 |  | 44,103,122 | 38,238,737 |  | 45,188,378 | 38,775,600 |  | 46,088,036 | 40,224,163 |
|  | 20-24 | 0·997 | 0·998 |  | 71·8 | 64·9 |  | 46,273,865 | 40,884,302 |  | 43,826,509 | 38,000,411 |  | 44,927,368 | 38,550,873 |
|  | 25-29 | 0·996 | 0·997 |  | 92·8 | 78·7 |  | 51,522,843 | 46,466,160 |  | 45,952,663 | 40,628,565 |  | 43,534,341 | 37,764,689 |
|  | 30-34 | 0·995 | 0·997 |  | 95·0 | 80·6 |  | 66,443,228 | 62,295,742 |  | 51,177,792 | 46,214,741 |  | 45,654,328 | 40,406,105 |
|  | 35-39 | 0·994 | 0·996 |  | 95·0 | 81·9 |  | 51,345,507 | 48,745,948 |  | 66,015,884 | 62,004,795 |  | 50,855,386 | 45,990,793 |
|  | 40-44 | 0·992 | 0·994 |  | 94·7 | 82·6 |  | 49,289,359 | 46,984,787 |  | 50,939,010 | 48,468,164 |  | 65,550,248 | 61,689,278 |
|  | 45-49 | 0·988 | 0·991 |  | 93·1 | 78·0 |  | 61,173,349 | 58,664,268 |  | 48,770,172 | 46,641,663 |  | 50,443,511 | 48,137,376 |
|  | 50-54 | 0·979 | 0·985 |  | 87·8 | 61·5 |  | 62,348,020 | 61,097,362 |  | 60,194,890 | 58,059,849 |  | 48,041,772 | 46,186,514 |
|  | 55-59 | 0·965 | 0·975 |  | 79·0 | 53·3 |  | 49,958,045 | 48,782,446 |  | 60,710,271 | 60,087,817 |  | 58,727,160 | 57,164,392 |
|  | 60-64 | 0·934 | 0·953 |  | 57·8 | 40·3 |  | 38,917,285 | 38,596,854 |  | 47,639,345 | 47,353,201 |  | 58,104,950 | 58,458,487 |
|  | 65-69 | 0·882 | 0·915 |  | 44·5 | 27·4 |  | 36,526,788 | 37,622,978 |  | 35,643,143 | 36,541,479 |  | 43,942,636 | 45,022,538 |
|  | 70-74 | 0·796 | 0·849 |  | 25·4 | 13·7 |  | 21,425,163 | 23,524,526 |  | 31,093,594 | 34,020,143 |  | 30,742,507 | 33,297,383 |
|  | 75-79 | 0·679 | 0·757 |  | 11·2 | 5·5 |  | 12,207,276 | 14,337,340 |  | 16,096,416 | 19,679,877 |  | 23,897,465 | 28,831,177 |
|  | 80+ | 0·568 | 0·624 |  | 11·2 | 5·5 |  | 10,587,091 | 16,031,012 |  | 12,511,382 | 18,984,646 |  | 16,333,051 | 24,919,303 |

**Table S3** Average annual direct expenditure and work loss days per colorectal cancer patient in China in 2019, by year post-diagnosis ^a^

| **Variable** | | **Number of**  **cases included** | **Direct medical expenditure (CNY)** | | |  | **Direct non-medical expenditure (CNY)** | | |  | **Work loss (Days)** | | |
| --- | --- | --- | --- | --- | --- | --- | --- | --- | --- | --- | --- | --- | --- |
|  |  |  | **Year 1** | **Year 2** | **Year 3** |  | **Year 1** | **Year 2** | **Year 3** |  | **Year 1** | **Year 2** | **Year 3** |
| Overall | | 2,356 | 60,732 | 55,124 | 48,487 |  | 5,395 | 4,925 | 5,084 |  | 81·4 | 90·2 | 74·1 |
| Age at diagnosis, years | |  |  |  |  |  |  |  |  |  |  |  |  |
|  | <45 | 332 | 9,051 | 9,999 | 6,687 |  | 933 | 970 | 513 |  | 83·5 | 143·3 | 85·4 |
|  | 45-59 | 917 | 8,999 | 7,466 | 5,844 |  | 891 | 631 | 705 |  | 93·6 | 69·6 | 64·6 |
|  | ≥60 | 1,107 | 8,512 | 8,037 | 7,774 |  | 883 | 734 | 800 |  | 67·9 | 98·1 | 77·7 |
| Gender | |  |  |  |  |  |  |  |  |  |  |  |  |
|  | Male | 1,345 | 8,774 | 7,376 | 5,331 |  | 875 | 648 | 666 |  | 83·5 | 77·0 | 66·6 |
|  | Female | 1,011 | 8,851 | 8,688 | 8,028 |  | 923 | 788 | 813 |  | 77·9 | 105·2 | 82·0 |
| Region | |  |  |  |  |  |  |  |  |  |  |  |  |
|  | East | 1,066 | 9,379 | 7,455 | 7,536 |  | 963 | 808 | 840 |  | 84·5 | 88·0 | 88·8 |
|  | Central | 643 | 8,623 | 8,330 | 9,776 |  | 1,087 | 494 | 707 |  | 95·6 | 168·8 | 41·8 |
|  | West | 647 | 8,084 | 9,214 | 5,166 |  | 708 | 557 | 523 |  | 70·6 | 66·9 | 53·1 |
| Stage | |  |  |  |  |  |  |  |  |  |  |  |  |
|  | I | 328 | 7,880 | 5,471 | 4,420 |  | 930 | 657 | 576 |  | 65·7 | 42·8 | 38·3 |
|  | II | 630 | 6,653 | 6,543 | 8,544 |  | 708 | 385 | 355 |  | 55·7 | 33·5 | 32·1 |
|  | III | 815 | 9,202 | 7,724 | 4,891 |  | 884 | 879 | 947 |  | 79·3 | 84·1 | 74·9 |
|  | IV | 559 | 10,049 | 8,822 | 8,063 |  | 1,013 | 724 | 765 |  | 104·2 | 113·5 | 91·1 |

^a^ To make sure the stability of annual expenditures, the analyzed sample for each year post-diagnosis should be over 50. Under this requirement, a total of 2356 colorectal cancer patients were included for the current analyses to produce the annual medical expenditure post-diagnosis, with the sample sizes for 1st to 3rd year being 803, 128, and 62, respectively. The literature showed that the expenditure of different locations of cancer occurred were almost the same, so we equated the expenditure and work loss days of colon, rectum, and anus cancer with the overall expenditure and work loss days. All expenditures expressed in 2019 Chinese Yuan (CNY)
